# Establishment and Application of PDCoV Antibody Indirect ELISA Detection Method Based on N Protein

**DOI:** 10.3390/vetsci13010012

**Published:** 2025-12-22

**Authors:** Yuting Xiao, Lei Zhou, Qin Gao, Ying Shan, Jidong Xu, Xiaoliang Li

**Affiliations:** 1 Department of Veterinary Medicine, College of Animal Sciences, Institute of Preventive Veterinary Medicine, Zhejiang University, Hangzhou 310058, China; yutingxiao@zju.edu.cn (Y.X.); 22317106@zju.edu.cn (L.Z.); 22017124@zju.edu.cn (Q.G.); shanying@zju.edu.cn (Y.S.); 2 The Rural Development Academy, Zhejiang University, Hangzhou 310058, China; 3 ZJU-Xinchang Joint Innovation Centre (TianMu Laboratory), Gaochuang Hi-Tech Park, Shaoxing 312500, China

**Keywords:** porcine deltacoronavirus (PDCoV), nucleocapsid protein, ELISA, antigen detection

## Abstract

Porcine deltacoronavirus (PDCoV) is a newly discovered porcine intestinal coronavirus that can pose a significant threat to the global commercial swine industry. We established an enzyme-linked immunosorbent assay (ELISA) detection method for the detection of PDCoV antibodies, based on the recombinant nucleocapsid (N) protein expressed using a baculovirus system. The assay was validated using positive and negative serum samples obtained from experimentally immunized rabbits and demonstrated an absence of cross-reactivity with either transmissible gastroenteritis virus (TGEV) or porcine epidemic diarrhea virus (PEDV). The recombinant PDCoV N protein antigen dilution (0.8 μg/mL), sample serum (1:400), and the enzyme-labeled secondary antibody (1:50) were used in this assay. The cut-off value was 0.355, without cross-reactivity including TGEV and PEDV. The ELISA method shows good sensitivity (96.67%), specificity (85.51%), and reproductivity (CV < 10%). We utilized the method to detect PDCoV antibodies in 600 pig serums collected from Zhejiang Province in the last four years (2021–2024). The results showed significant differences in antibody levels between regions and considerable fluctuation in positivity rates across the four-year period. As shown in the results, we developed a sensitive and specific ELISA method for detecting anti-PDCoV N antibodies, which provides a rapid and reliable diagnostic tool for PDCoV surveillance and control. This assay demonstrates significant potential for both epidemiological investigations and commercial applications in swine disease management.

## Simple Summary

A newly emerged pig virus, porcine deltacoronavirus (PDCoV), poses a serious threat to swine farms worldwide. To help control its spread, we developed a novel, efficient blood serum test to detect if a pig has been infected. This test works by identifying antibodies against the virus in a pig’s blood. Our method is confirmed to be accurate and did not confuse PDCoV with other viruses causing pig diarrhea. By testing 600 pig serum samples within Zhejiang Province over four years, we found that the infection rates varied significantly between different areas and from year to year. This new test provides a rapid and reliable tool for veterinarians and farmers to monitor the virus, helping to protect pig herds and support the swine industry.

## 1. Introduction

Porcine deltacoronavirus (PDCoV) is a newly discovered porcine intestinal coronavirus that causes vomiting, diarrhea, dehydration, weight loss, lethargy, and death in piglets [1,2]. PDCoV was initially identified in porcine rectal swab specimens in Hong Kong in 2012 and designated as HKU 15 coronavirus. This was followed by an initial outbreak affecting multiple pig farms in Ohio, United States, in 2014 [1,2]. Subsequently, cases of the disease were also discovered in several other countries, including South Korea, Thailand, Laos, Vietnam, Mexico, and China [3,4,5,6,7,8,9]. This rapid and widespread spread of PDCoV has taken a clear toll on the global swine industry. PDCoV, a member of the genus *δ-Coronaviruses* of the family *Coronaviridae*, is the smallest known single-stranded positive-RNA virus [1]. Its genome structure is 5′UTR-ORF1a-ORF1b-S-E-M-NS6-N-NS7-3′UTR, which is similar to other coronaviruses [10,11]. The whole viral genomic RNA encodes four structural proteins, including the spike (S) protein, the envelope (E) protein, the membrane (M) protein, and the nucleocapsid (N) protein [12]. N proteins are located in the nucleolus and are involved in the assembly of the nucleoshell, viral RNA synthesis, virus–host interactions, and various cellular processes including metabolism, stress response, protein biosynthesis and transport, and cell division [13,14]. Although the N protein and the other three structural proteins play a crucial role in the life cycle of PDCoV, the N protein of PDCoV has the most conserved sequence among different strains compared to other structural proteins, making it a better choice for antigen detection [15].

At present, PDCoV diagnostic methods can be divided into two categories: etiology and serology. Etiological detection methods can directly detect the presence and virus titer, and common detection methods include virus particle detection, reverse transcription-PCR (RT-PCR), and quantitative real-time RT-PCR (qRT-PCR) [10,16,17,18]. Serological assays directly detect the host’s immune response to PDCoV. They are thus frequently employed to evaluate vaccine immunogenicity and investigate host immune response mechanisms. Widely used serological methods include indirect immunofluorescence assay (IFA), virus neutralization (VN), fluorescent multiplexed bead-based immunoassay (FMIA), and enzyme-linked immunosorbent assay (ELISA) [12,19,20]. Compared with other serological assays, ELISA has lower instrument requirements, simple test steps, shorter cycles, low cost, and good portability and can distinguish between different antibody types and kits with viral structural proteins as antigens have no risk of virus transmission. In addition, the ELISA method is capable of large-scale clinical sample detection, which is more suitable for rapid and efficient PDCoV diagnosis.

Depending on the purpose of the detection, ELISA detection methods can be divided into antigen-specific and antibody-specific detection methods. Indirect ELISA and blocking ELISA are the most often employed among the total ELISA detection methods. At present, several ELISA antibody detection methods based on the PDCoV S protein, M protein, and N protein have been established. Although the S protein has good antigenicity, it is an essential structural protein for inducing neutralizing protective antibodies and is a better choice for antigens in the ELISA detection method. With the relatively lower expression level leading to a higher cost for the ELISA detection method, the S protein cannot be used for large-scale production [21,22]. With the small molecular weight, the PDCoV M protein is much easier for recombinant expression than the S protein; while cross-reactivity of PDCoV with antibodies against the transmissible gastroenteritis virus (TGEV) and porcine epidemic diarrhea virus (PEDV) M protein has not been observed, no detection systems have been established for the M protein [10,23]. The N protein is the highest expressed viral protein during PDCoV infection, which stimulates host cells to produce high levels of PDCoV-specific antibodies. In addition, the N protein is highly conserved within the PDCoV structural and non-structural proteins, thus it is the better choice for developing a PDCoV N protein-based detection method among the ELISA detection methods for PDCoV [19,24,25].

In recent years, most ELISA assays for PDCoV antibody detection have plate-coated antigens from mammalian cell expression systems or *E. coli* expression systems [12,26]. The mammalian expression system is limited by its high cost and low protein yield, making large-scale production challenging. However, the lack of post-translational modifications in eukaryotic cells in the proteins expressed by *E. coli* results in a large difference in function and conformation between the expressed proteins and native proteins, which affects the sensitivity and specificity of ELISA [27].

Therefore, there is an urgent need to develop an ELISA method with high sensitivity, high specificity, and practical application, to provide a rapid method for the identification of PDCoV in the pig industry. In this study, we have established an indirect ELISA detection method for the detection of PDCoV by utilizing the recombinant PDCoV N protein prepared by the Bac-to-Bac baculovirus expression system as the coating antigen and the PDCoV antibody-positive serum and -negative serum prepared from the experimental rabbits. After the reaction conditions, if the ELISA method was optimized, we evaluated the quality (including the sensitivity, reproducibility, specificity, etc.) of the indirect ELISA detection method for PDCoV antibodies. Finally, we detected the PDCoV antibodies in 600 clinical porcine sera from different regions of Zhejiang Province, China. Therefore, the ELISA method established herein is suitable for the epidemiological surveillance of PDCoV and serves as a rapid, effective tool for its prevention and control, demonstrating potential for commercial application.

## 2. Materials and Methods

### 2.1. Cells, Viruses, Experimental Animals, PDCoV Strain, Inactivated Vaccine and Serum, and Clinical Samples

The Sf9 cell line, Vero cell line, and LLC-PK1 cell lines were stored in our laboratory. Sf9 cells were cultured in Sf-900™ II SFM (Thermo Fisher Scientific, Waltham, MA, USA) medium at 27 °C (without CO_2_). Vero cells were cultured in Dulbecco’s Modified Eagle Medium (DMEM; Gibco, Grand Island, NY, USA) supplemented with 10% fetal bovine serum (FBS; Gibco) and 1% antibiotic–antimycotic (Gibco), maintained in a 5% CO_2_ incubator at 37 °C. The LLC-PK1 cells were cultured in Dulbecco’s Modified Eagle Medium-F12 (DMEM-F12; Gibco) with 10% FBS (Gibco) and 1% antibiotic–antimycotic and grown in a 5% CO_2_ incubator at 37 °C.

PDCoV ZJ17DQ0301 strain was isolated from a pig farm in Deqing County, Zhejiang Province, China, and stored in our laboratory at −80 °C [28].

Health experimental rabbits were purchased from Hongfeng Rabbit Farm in the Fuyang district of Hangzhou, Zhejiang Province; the PDCoV-inactivated vaccine was provided by the Jiangsu Academy of Agricultural Sciences. Pig anti-PDCoV and pig anti-TGEV-positive serum were provided by the Jiangsu Academy of Agricultural Sciences. The following positive serums were obtained from our laboratory: rabbit anti-pig PDCoV-, pig anti-PEDV-, TGEV-, PRRSV-, CSFV-, and PCV2-positive serum. PDCoV N monoclonal antibody 2H7 was developed and preserved by our laboratory.

Clinically healthy, unvaccinated 2–7-day-old piglet serum samples were provided by the Chia Tai Pig Industry (Yuyao) Co., Ltd., Yuyao, China. The sera were isolated from different regions of Zhejiang Province between the years 2018 and 2024.

### 2.2. Recombinant Plasmid Construction and Transfection

The recombinant plasmid pFastBac1-PDCoV-N-9×His, preserved in our lab, was PCR-amplified using PDCoV-N primers (PDCoV-N-F: TGCACCAGTAGTCCCTACTA, PDCoV-N-R: CGCTGCTGATTCCTGCTTTA), to demonstrate a single band of expected size, followed by sequence confirmation (Hzykang Biotech, Hangzhou, China). Following sequence confirmation, the plasmid was transformed into DH10Bac competent cells. The recombinant clones were screened by blue–white selection, and positive colonies were verified by PCR amplification using pUC primers (pUC-F: CCCAGTCACGACGTTGTAAAACG, pUC-R: AGCGGATAAC AATTTCACACAGG). Colonies showing a single band of expected size were sequenced, and those with correct sequence alignment were preserved as glycerol stocks (designated Bac-PDCoV N-His) at −80 °C. The recombinant baculovirus plasmid was extracted using the SanPrep Column Plasmid Mini-Preps Kit (Sangon, Shanghai, China), PCR-verified with PDCoV-N and pUC upstream and downstream primers, and stored at −20 °C after verification. Afterwards, the recombinant bacmids and Cellfectin™ II Reagent (Thermo, Waltham, MA, USA) were diluted in Sf-900™ II SFM, mixed after 20 min incubation, and transfected into Sf9 cells at 27 °C. After 3–5 h post transfection, the medium was replaced with fresh Sf-900™ II SFM. After 5 days, the culture was centrifuged (1000× *g*, 10 min) to collect baculovirus supernatant and cells.

### 2.3. PDCoV N Protein Recombinant Expression

Initially, the recombinant PDCoV N protein was expressed using the Bac-to-Bac system. After recombinant virus infection, transfection efficiency was detected by IFA (the detailed method is below), showing specific fluorescence in the infected Sf9 cells. Primary viral stocks (P1) were aliquoted and stored at −80 °C. P2 and P3 stocks (3–5 days post-infection, 27 °C) were sequentially amplified using Sf-900™ II SFM (10%, FBS) in T75 flasks (70–80% confluence), collected by centrifugation (4000× *g*, 5 min) for each passage, and stored at −80 °C. Next, Sf9 cells were infected with P3 viral stock and cultured in suspension at 27 °C with shaking. At 72 h.p.i., the culture was centrifuged (4000× *g*, 5 min) to separate the supernatant and cell pellet. The recombinant protein was predominantly present in the infected cell pellet. The pellet was resuspended and lysed by sonication, followed by centrifugation (12,000× *g*, 20 min) to clarify the lysate. The supernatant was subjected to HisSep Ni-NTA Agarose Resin (Yeasen Biotechnology, Shanghai, China) with imidazole (Thermo) gradient elution. Elution fractions containing recombinant protein (>0.2 mg/mL as determined by BCA assay; Beyotime, Shanghai, China) were pooled following Western blotting (detailed protocol shown below) confirmation and stored at −80 °C for downstream applications as a coating antigen in ELISA.

### 2.4. Positive and Negative Serum Preparation

Experimental rabbits were immunized via subcutaneous multi-point injections with either inactivated PDCoV vaccine (positive control) or DMEM medium (negative control) at days 0, 7, and 21 (0.5 mL per injection). At day 35 post-immunization, serum was collected by cardiac puncture, clarified by centrifugation, sterilized by filtration, heat-inactivated (56 °C for 30 min), and stored at −80 °C. Anti-PDCoV N antibody titers were quantified by ELISA: 96-well plates were coated with recombinant N protein overnight at 4 °C, blocked with ELISA buffer (37 °C, 1 h), then incubated with 400-fold diluted serum samples (collected days 0–35 post-immunization and per 7 days). After PBST washes, HRP-conjugated SPA (1:10,000) was incubated at 37 °C for 1 h. After final washes, TMB substrate (pre-mixed A:B = 1:1) was added and incubated at 37 °C for 15 min, reactions were terminated with 2M H_2_SO_4_, and OD_450nm_ was measured. Serum anti-PDCoV N antibodies were subsequently verified by IFA. LLC-PK1 cells were infected with a 5000-fold dilution of PDCoV strain ZJ17DQ0301 and incubated at 37 °C for 48 h and confirmed by IFA.

### 2.5. Cross-Reactivity Detection of Three Porcine Intestinal Coronavirus N Proteins

For expression of the required exogenous virus and the control proteins, recombinant plasmids, pCMV-PEDV N-HA, pCMV-PDCoV N-HA, pCMV-TGEV N-HA, and pCMV-HA, were transfected into the Vero cells using HighGene transfection reagents (ABclonal, Woburn, MA, USA) according to the manufacturer’s instructions. After 24 h post transfection, the cross-reactivity was detected by Western blotting and IFA, and the detailed methods are described below.

### 2.6. Optimization of ELISA Method

The ELISA conditions were systematically optimized in the 96-well coated with high-binding polystyrene, including testing PDCoV-N protein coating concentrations (0.4–2.8 μg/mL), serum dilution ratios (1:100–1:800), and coating durations (10/14/18 h at 4 °C). Additional optimizations included determining the optimal blocking time (0.5–2.5 h), serum incubation period (40–70 min), HRP-rec-Protein A secondary antibody (1:5000, 1:10,000, 1:20,000) incubation time (30–60 min), and TMB substrate development duration (5–20 min) to establish the optimal assay conditions. The P/N values of each group were compared under the above optimization conditions, and the maximum P/N value was selected as the corresponding optimal condition.

### 2.7. Analysis of ROC Curve of ELISA

A total of 543 clinical porcine serum samples were tested by IFA and ELISA. The sample-to-positive (S/P) ratio was calculated using the following formula: S/P = (OD_450nm_ sample − OD_450nm_ negative control)/(OD_450nm_ positive control − OD_450nm_ negative control). The S/P values were compared with the IFA results as the reference standard. Receiver operating characteristic (ROC) curve analysis was performed using GraphPad Prism 9 software. The optimal cut-off value, along with corresponding sensitivity and specificity, was determined by maximizing the Youden index.

### 2.8. Assessment of Reproducibility and Specificity of ELISA Method

To evaluate the reproducibility of this method, we were performed two assays. One was an intra-batch reproducibility test: recombinant PDCoV N protein was coated for 1–3 days, with one plate selected daily. A total of16 randomly selected clinical serum samples were then tested per plate. The other was the inter-batch reproducibility test: the recombinant PDCoV N protein was coated at different time periods and 16 serums were detected. The coefficient of variation (CV) was used to determine the reproducibility of the intra-batch and inter-batch with OD_450nm_ values. At the same time, PEDV-, TGEV-, PRRSV-, CSFV-, and PCV2-positive serums were detected, and the specificity of the method was analyzed by S/P value.

### 2.9. Validation with Clinical Samples

To evaluate the performance of PDCoV antibody ELISA on clinical samples, 600 serum samples were tested for PDCoV from pig farms in different regions of Zhejiang Province, China. Each serum sample was tested in triplicate. The detailed detection steps are shown in the Section 2, and the optimization of the conditions of the method is shown in the results below.

### 2.10. Western Blotting

Cells were washed twice with Hank’s Balanced Salt Solution (Beyotime) and lysed in RIPA buffer (Beyotime) for total protein extraction. Protein concentration was quantified using a BCA assay kit (Beyotime). To detect the expression level of the required proteins, Western blotting was performed as below, based on a previous study [29]: Aliquots containing 20 µg of total protein were mixed with SDS-PAGE loading buffer, heat-denatured, and resolved on 12% SDS-polyacrylamide gels. Proteins were electrophoretically transferred to a 0.22 μm polyvinylidene difluoride membrane (PVDF, Millipore, Darmstadt, German). Membranes were blocked with 5% skim milk (Sangon) in TBST (Sangon) for 1 h at room temperature, followed by sequential incubation with primary and secondary antibodies as specified below: pig anti-PEDV-positive serum, pig anti-TGEV-positive serum, and rabbit anti-pig PDCoV-positive/negative serum (1:200), and His Tag Mouse Monoclonal Antibody (Beyotime) as primary antibodies and HRP-labeled goat anti-mouse IgG(H+L) (Beyotime) Alexa Fluor 555 donkey anti-mouse IgG (Invitrogen, Waltham, MA, USA) as secondary antibodies. Finally, the protein bands were visualized using the Imaging System (SageCreation, Beijing, China).

### 2.11. IFA

To visualize and detect PDCoV antibodies in porcine serum, an immunofluorescence assay was performed as per a previous study [30]. Vero cells were fixed with 4% paraformaldehyde (Beyotime) at room temperature for 40–60 min, followed by permeabilization with 0.1% Triton X-100 (Sangon) in PBS for 20 min. Then samples were incubated with primary antibodies for 1 h as follows: pig anti-PEDV-positive serum, pig anti-TGEV-positive serum, rabbit anti-pig PDCoV-positive serum, and pig anti-PDCoV-positive serum (1:50), then with secondary antibodies for 1 h at 37 °C in the dark: Alexa Fluor 488 goat anti-rabbit IgG (Invitrogen; 1:1000) and Alexa Fluor 555 donkey anti-mouse IgG (Invitrogen; 1:1000). Nuclei were labeled with DAPI (Thermo; 1:1000) for 5 min. Images were acquired using a fluorescence microscope (Olympus, Tokyo, Japan).

## 3. Results

### 3.1. Induction of Expression and Purification of Recombinant Proteins

**Figure 1:**
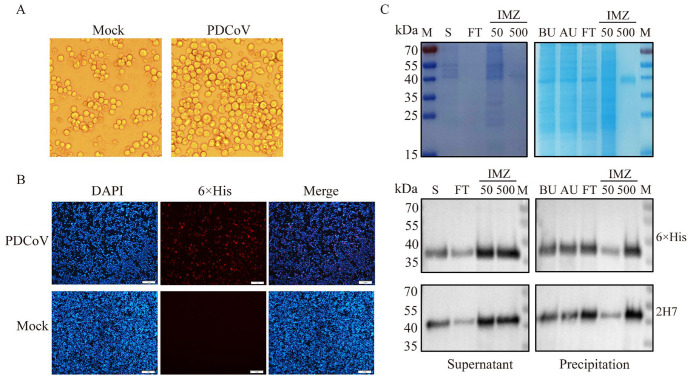
Expression of recombinant PDCoV N protein. (**A**): Pathological changes in Sf9 cells infected with recombinant bacmid. Observations under the microscope revealed that cells in the P1 generation displayed characteristic pathological features, including rounding, enlargement, and significant swelling. (**B**): Verification of PDCoV N protein expression by IFA. Using IFA detection, no fluorescence was observed in the control group of Sf9 cells, whereas specific red fluorescence was detected in the majority of cells infected with the recombinant virus. Scale bar = 1 mm. (**C**): The supernatant and precipitate of the recombinant PDCoV N protein were detected by SDS-PAGE and Western blot. The protein precipitate was resuspended in 0.01 mM PBS and sonicated for 30 min, followed by centrifugation to collect the supernatant. The target protein was eluted with 500 mM imidazole and detected by the 12% SDS-PAGE. The Western blot analysis revealed distinct bands at 42 kDa in both the supernatant and the purified precipitate samples. The primary antibodies are 6×His mouse monoclonal antibody and PDCoV N mouse monoclonal antibody 2H7. M: Marker; S: Supernatant; FT: Fluid penetration; IMZ: Imidazole; BU: Before Ultrasound; AU: Supernatant obtained by ultrasonic centrifugation.

To express the recombinant PDCoV N protein, the constructed recombinant bacmid Bac-PDCoV N-His was transfected into Sf9 cells. Microscopic observation revealed characteristic cytopathic effects in P1-infected cells, including cell rounding, enlargement, and significant swelling, indicating successful baculovirus infection (Figure 1A). No fluorescence was observed in the control cells of Sf9 cells detected by IFA, while specific red fluorescence was observed in most cells infected with the recombinant virus (Figure 1B). SDS-PAGE results demonstrated that the recombinant PDCoV protein, exhibiting the expected size of approximately 42 kDa, was expressed in both the supernatant and precipitation fractions. Following purification using nickel affinity chromatography, SDS-PAGE results showed the obtained recombinant PDCoV N protein was of high purity (purity percent of 96.6%), and Western blotting analysis confirmed the presence of a specific 42 kDa band. This protein of 42 kDa molecular weight was exclusively recognized by both a 6×His mouse monoclonal antibody (Beyotime) and the PDCoV N mouse monoclonal antibody 2H7 made in our laboratory, without cross-reactivity observed with other bands (Figure 1C, Figure S1).

### 3.2. Preparation of PDCoV Antibody-Positive and -Negative Control Serum

**Figure 2:**
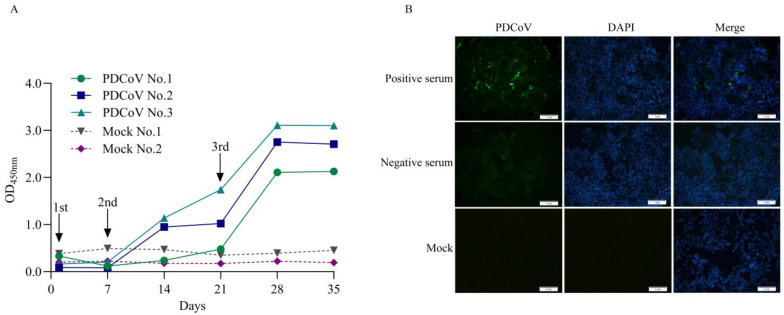
Preparation of anti-PDCoV N antibody-negative and -positive serum. (**A**): Anti-PDCoV N antibody levels during immunization. The levels of anti-PDCoV antibodies in rabbit sera collected at different times were detected using the laboratory indirect ELISA method. After three immunizations, the antibody levels in the experimental rabbits all significantly increased. (**B**): The IFA validation results of positive and negative control serum. LLC-PK1 cells infected with PDCoV strain ZJ17DQ0301 for 48 h were subjected to IFA using negative and positive rabbit control serum (1:200) as primary antibodies, followed by an FITC-conjugated goat anti-rabbit secondary antibody (1:1500). Scale bar = 1 mm. Green fluorescence represents PDCoV antigen binds to specific antibodies in the serum and blue fluorescence represents cell nuclei (DAPI).

The level of Anti-PDCoV N antibody in serum collected after immunization (0, 7, 14, 21, 28, and 35 days) was detected using the commonly used indirect ELISA detection system and the recombinant PDCoV N protein prepared in this experiment. The results showed that the antibody levels of experimental rabbits No. 1 increased significantly after the three immunizations, and the antibody levels of experimental rabbits No. 2 and No. 3 gradually increased after the second immunization on the seventh day and increased significantly after the three immunizations, which was in line with the laws of immunology. However, there was no significant change in the antibody level of PDCoV N in the serum of rabbits in the control group from 0 to 35 days (Figure 2A). Also, the results of IFA showed that the rabbit positive serum specifically bound to LLC-PK1 cells infected with PDCoV, and strong green fluorescence was observed. However, the negative serum could not bind to PDCoV and could not be observed with green fluorescence (Figure 2B). The results indicated that the positive and negative control serum was successfully prepared.

### 3.3. Analysis of the Cross-Reactivity of Porcine Intestinal Coronavirus N Proteins

**Figure 3:**
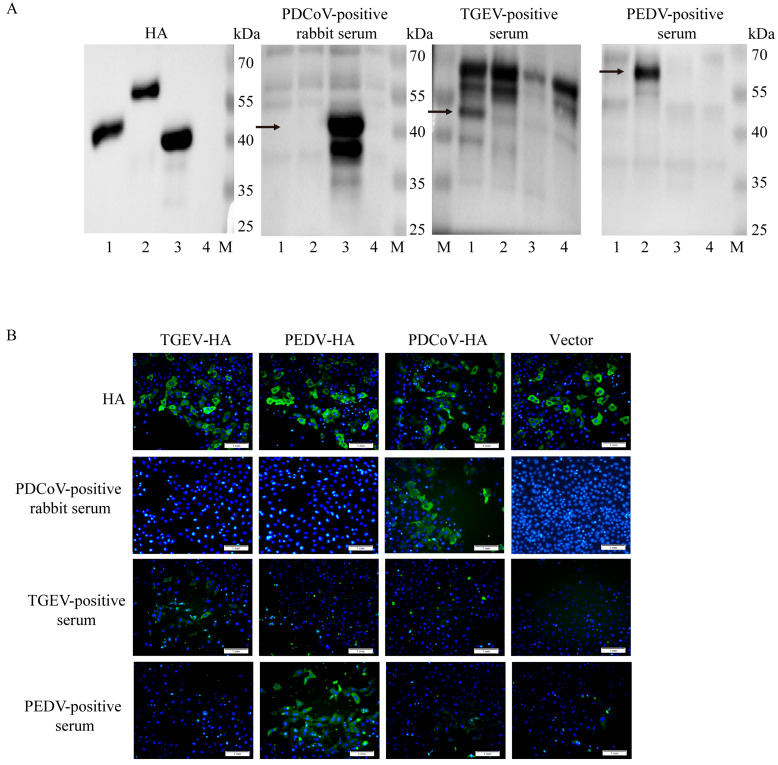
Cross-reactivity analysis of three porcine intestinal coronavirus N proteins. (**A**): Western blot analysis was performed using the N proteins of three porcine enteric coronaviruses and control proteins as antigens. M: Marker; line 1: TGEV-N-HA plasmid; line 2: PEDV-N-HA plasmid; line 3: PDCoV-N-HA plasmid; line 4: HA control plasmid. Arrows are the molecular weights of target bands. (**B**): The cross-reactivity test results of the N proteins of three porcine intestinal coronavirus strains were obtained by IFA. Overexpression of the N protein of three porcine intestinal coronavirus strains, with 5 replicates well set for each protein. IFA detection was performed 24 h later. The antibodies are rabbit anti-pig PDCoV-positive serum, pig anti-TGEV-positive serum, and pig anti-PEDV-positive serum. All three positive serums could only recognize the N protein of their respective pathogens, without any cross-reaction. Scale bar = 1 mm. Green fluorescence represents specific recognition of the homologous N protein by positive serum and blue fluorescence represents cell nuclei (DAPI).

The pCMV plasmid was successfully used to overexpress the HA-tagged N proteins of TGEV, PEDV, and PDCoV in Vero cells with a single band of interest and the expected size (Figure 3A, Figure S2). TGEV-, PEDV-, and PDCoV antibody-positive rabbit serum only recognized the N protein of the corresponding pathogen, and there was no reactivity. The IFA assay results demonstrated that specific green fluorescence of the cell shape could be observed under fluorescence microscopy after incubation of cells with HA tags (Figure 3B). TGEV-, PEDV-, and PDCoV antibody-positive rabbit serum can only specifically bind to their corresponding pathogenic N proteins and emit specific green fluorescence after being conjugated by fluorescent secondary antibodies.

### 3.4. Establishment of Indirect ELISA Method and Optimization of Conditions

#### 3.4.1. Optimization of Antigen Coating Concentration and Serum Dilution

**Figure 4:**
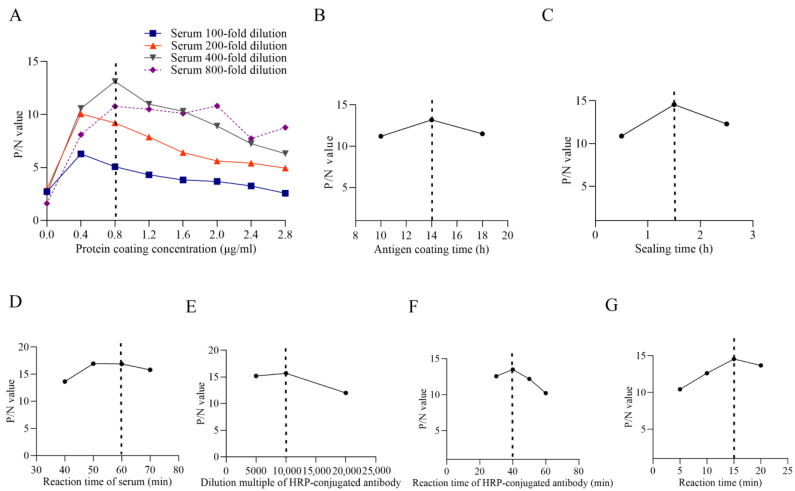
Optimization for indirect ELISA. (**A**): Optimization of the protein coating concentration. (**B**): Optimization of the antigen coating time. (**C**): Optimization of the sealing time. (**D**): Optimization of the reaction time of the serum. (**E**): Optimization of the dilution multiple of HRP-conjugated antibody. (**F**): Optimization results of reaction time of HRP-conjugated antibody. (**G**): Optimization results of reaction time of developer. The dotted lines within the figures present the optimized value.

The optimized ELISA conditions were determined as follows: antigen coating concentration at 0.8 µg/mL; serum dilution at 1:400; antigen coating time of 14 h; blocking time of 1.5 h; serum incubation time of 1 h; HRP-conjugated secondary antibody dilution at 1:10,000 with an incubation time of 40 min; and substrate incubation time of 15 min (Figure 4A–G).

#### 3.4.2. Determination of the Cut-Off Value of ELISA

**Figure 5:**
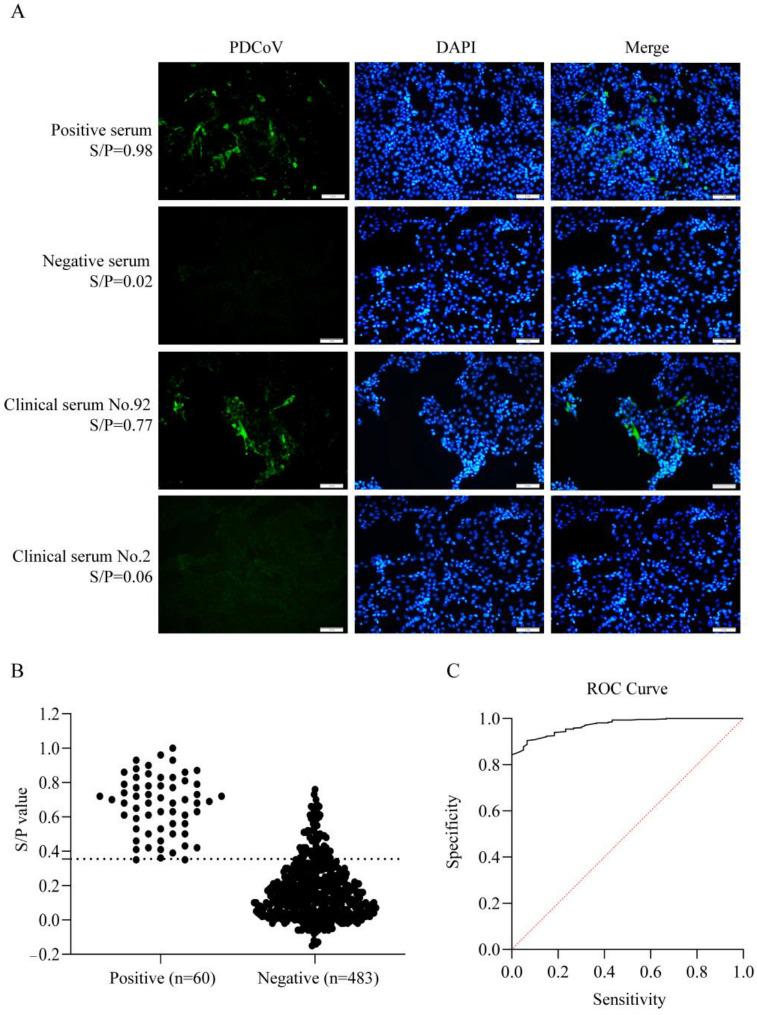
Determination of the cut-off value of ELISA. (**A**): IFA results of positive and negative control serum and two clinical samples. (**B**): Data summary of IFA and ELISA results. The dashed line indicates the optimal cut-off value. (**C**): The results of ROC curve analysis. The red line represents the reference line.

Using IFA as the gold standard, we tested 543 clinical pig serum samples (Figure 5A), identifying 60 positive (11.0%) and 483 negative (89.0%) samples. These IFA results served as the reference to evaluate the diagnostic performance of our ELISA method. For ELISA, we measured OD_450nm_ values of all clinical serum and calculated S/P ratios (IFA test positive = 1, negative = 0); the S/P ratios obtained by the two methods were compared (Figure 5B). A *p* value < 0.0001 indicates high statistical significance, while an area under the curve (AUC) value of 0.9709 confirms the high diagnostic accuracy of this ELISA method. The optimal cut-off value was 0.355, and the sensitivity and specificity were 96.67% and 85.51%, respectively (Figure 5C).

### 3.5. Analysis of the Reproducibility and Specificity of ELISA

Based on the optimized ELISA conditions established above, we evaluated the stability and specificity of the method. In the intra-batch repeatability test, the serum CV ranged from 0.82 to 9.11% (Table 1). In the inter-batch repeatability test, the CV of the serum ranged from 2.66 to 9.65%, all of which were less than 10% (Table 2). In addition, the specificity test proved that the positive serums of PDCoV, PEDV, TGEV, CSFV, PCV2, and PRRSV were detected simultaneously using this method, and the results were all judged to be negative except for the positive serum test results of PDCoV, which could be judged to be positive without cross-reactivity (Table 3).

### 3.6. Clinical Samples Detection by ELISA

**Figure 6:**
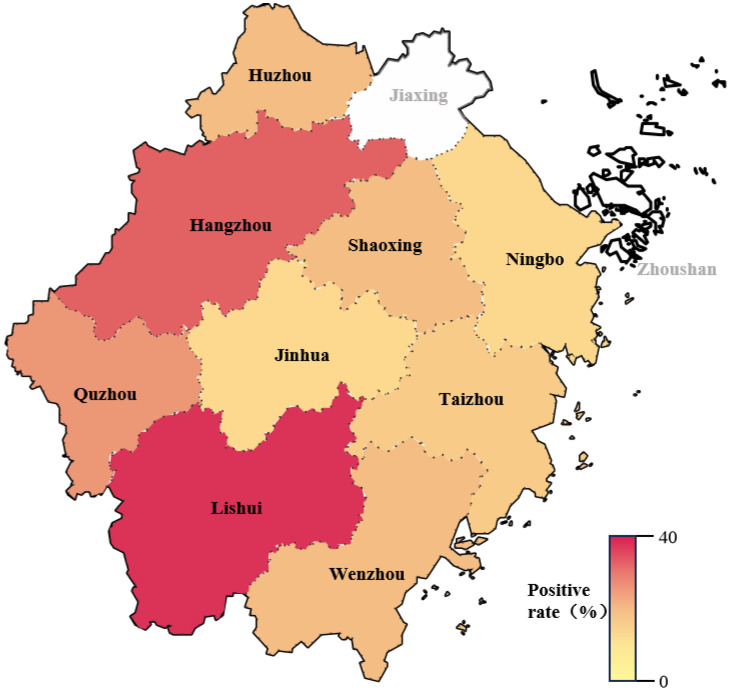
Positive rate of PDCoV antibody in pig herds from different regions in Zhejiang province in the past four years. The white sections indicate that no clinical samples were collected in Jiaxing and Zhoushan.

The PDCoV-N antibody ELISA was tested on 600 serum samples from pig herds in nine regions of Zhejiang Province from 2021 to 2024. The result showed that the overall seroprevalence was 19.3% (116/600). Regional analyses revealed significant differences. The positive rate was the highest in Lishui (42.7%), followed by Hangzhou (30.3%) and Quzhou (21.2%), while the positive rates in the other six regions were lower, ranging from 7.5% to 15.3% (Figure 6 and Table 4, Table 5 and Table 6).

## 4. Discussion

Porcine deltacoronavirus (PDCoV) infection poses a great threat to piglet health, with a mortality rate of 30–40%, which seriously affects the healthy breeding of pigs [31]. The N protein of PDCoV binds to viral RNA to form the nucleocapsid and is highly abundant, immunogenic, and specific, inducing strong IgG antibody production in hosts [15]. Among the PDCoV proteins (N, S1, and M), the N protein is the most conserved, with 96.9–100% amino acid homology, making it the best antigen for ELISA detection. Therefore, due to its stability and high immune response, the N protein is preferred to the S1 and M proteins as diagnostic targets [25].

The purity, stability, and post-translational modifications of the coated antigen have a significant impact on the ELISA method. Currently, PDCoV antibody ELISAs primarily use antigens from mammalian or *E. coli* expression systems. In 2016, Su et al. developed an indirect ELISA detection method based on prokaryotic expression of the PDCoV N protein, which has a sensitivity of up to 100%, a specificity of 90.4%, and reacts exclusively with PDCoV [24]. Okda et al. established both an indirect ELISA and fluorescent microsphere immunoassay (FMIA) for PDCoV antibody detection using prokaryotically expressed N protein, demonstrating high diagnostic accuracy (96.1% sensitivity and 96.2% specificity), and it did not react with other related porcine pathogens [19]. In this study, we developed an improved approach using the Bac-to-Bac insect–baculovirus system to express recombinant PDCoV N protein, which provides higher yields (up to 50% of cellular protein) and maintains proper post-translational modifications for native protein structure [32,33,34]. Also, it can offer enhanced biosafety through the nonpathogenic vertebrate Autographa californica nucleopolyhedrovirus (AcMNPV) [35]. The high-purity antigen produced showed excellent performance in ELISA, establishing a superior diagnostic reagent for PDCoV serological detection that overcomes the limitations of conventional expression systems.

PDCoV infection causes clinical symptoms nearly identical to TGEV and PEDV, requiring laboratory tests for accurate diagnosis [36]. However, due to the high conservation of coronavirus N proteins, cross-reactivity among these three viruses may complicate serological detection [37]. The study confirmed that the PDCoV N protein shows no cross-reactivity with TGEV or PEDV antibodies, supporting its specificity for PDCoV diagnosis, consistent with the findings of Lin et al. [38]. Simultaneously, the rabbit anti-PDCoV-positive serum prepared in this experiment also did not cross-react with the N proteins of TGEV and PEDV, which was in line with expectations. To obtain serum that is not contaminated with other porcine pathogens and to ensure the reproducibility of control serum production, rabbits are immunized to produce large amounts of rabbit anti-PDCoV serum. Therefore, it is necessary to select enzyme-labeled secondary antibodies that can simultaneously recognize multiple animal antibodies. Moreover, the use of recombinant HA-tagged N proteins enables sensitive and controlled detection of cross-reactive epitopes, revealing significant serological cross-reactivity among PDCoV, PEDV, and TGEV. However, this approach may not fully mimic the native conformational antigenicity of N proteins within authentic virions or infected host cells. Future studies with whole-virus antigens or in vivo models are needed to confirm the biological relevance of these findings. Protein A is found on the surface of *Staphylocaux aureus* and can bind to the Fc fragment of IgG in a variety of mammals (pigs, human, rabbits, etc.). In addition, protein A contains five different domains that can bind to IgG Fc fragments, and each protein A molecule can bind to at least two IgG molecules with strong affinity [39]. So, HRP-rec-Protein A was selected as the enzyme-labeled secondary antibody in this method. To establish the optimal cut-off value (negative and positive serum), 543 clinical pig sera were tested by ELISA and IFA, with ROC analysis determining 0.355 as the optimal value. The method demonstrated high specificity and repeatability, as evidenced by the absence of cross-reactivity with five porcine pathogens (including PEDV and TGEV) and by all inter-batch and intra-batch CVs being below 10%. Finally, our clinical porcine serum sample testing using the developed ELISA detection method showed that there were significant regional differences in the PDCoV seropositivity rate in Zhejiang Province from 2021 to 2024, with an overall antibody positivity rate of 19.3% (116/600). As shown in Table 5 and Figure 6, the positive rate of the regions Lishui and Hangzhou was significantly higher than that of other areas. The highest seropositivity rates were clustered in western Zhejiang (Lishui 42.7%, Hangzhou 30.3%, and Quzhou 21.2%), a geographical pattern potentially linked to the high prevalence (33.7%) reported in the adjacent Jiangxi Province by Song et al. using RT-PCR detection in 2015, suggesting possible cross-regional transmission dynamics [9]. This might be attributed to potential factors such as different farming density, environmental conditions, or biosecurity measures taken in different cities. Specifically, Hangzhou relies heavily on live pigs transported from other regions, rather than local production. This creates a major risk, as animals from multiple external sources mix. This complex supply chain may increase the pressure of disease prevention, leading to a higher detection rate of viruses like PDCoV. On the other hand, livestock farming in Lishui is characterized by a large number of small-scale, scattered households; this structure often results in inadequate implementation of biosecurity measures, which may also lead to a high positive rate of PDCoV. In contrast, Ningbo has a higher proportion of large-scale, industrialized pig farms; the administrations are relatively mature and strict in porcine health management and epidemic surveillance. This rigorous monitoring and control is a key factor leading to the lower detection rate of PDCoV within this region. Furthermore, the number of serum samples included in this study is still small, and the samples from Jiaxing and Zhoushan are not covered. This may be affected by regional breeding policy adjustments and local economic development transformation of Jiaxing and Zhoushan, leading to the industrial structure of pig farming undergoing significant changes. As a result, the proportion of the traditional self-reproduction and self-raising model has continued to decline, directly leading to a reduction in the scale of pig breeding in both areas. With significantly fewer farms and pigs available, surveillance sampling could not reach suitable sources, resulting in no effective samples being collected. Thus, further serum will be collected in the future to increase the number of tests to obtain a more reliable PDCoV antibody-positive rate.

It is also worth mentioning that serological ELISA alone exhibits inherent limitations for precise PDCoV surveillance, primarily due to its inability to differentiate between active infection and prior exposure or to indicate viral shedding status. Serological assays provide an efficient means to evaluate herd-level immunity and infection pressure. Following the identification of seropositive or clinically suspect animals, confirmatory testing of fecal samples via RT-PCR or analogous molecular methods is essential to verify active infection, characterize circulating strains, and quantify viral load. This combined methodological approach yields the comprehensive dataset required to inform targeted disease control interventions.

In summary, the developed ELISA detection method represents an efficient and cost-effective diagnostic platform for high-throughput PDCoV antibody detection, demonstrating excellent stability, specificity, and sensitivity. Also, ELISA testing has a wide range of applications, not only to detect antibody levels in animals and evaluate the prevalence and vaccination effect of diseases but also to directly detect antigens. More importantly, our developed ELISA detection method can be used as a practical tool for epidemiological investigations in resource-limited settings. The development of this reliable ELISA has great potential for commercialization, which will support PDCoV prevention and control strategies and ultimately support the sustainable development of the swine industry.

## Figures and Tables

**Table 1:** Detection results of intra-batch repeatability.

Number	Days			Mean	SD (%)	CV (%)
	1	2	3			
1	0.791	0.809	0.769	0.790	2.00	2.54
2	0.073	0.065	0.078	0.072	0.66	9.11
3	1.606	1.579	1.589	1.591	1.37	0.86
4	0.114	0.117	0.109	0.113	0.40	3.57
5	0.061	0.056	0.059	0.059	0.25	4.29
6	0.082	0.093	0.087	0.087	0.55	6.31
7	0.093	0.109	0.099	0.100	0.81	8.06
8	0.175	0.174	0.170	0.173	0.26	1.53
9	0.314	0.313	0.307	0.311	0.38	1.22
10	0.096	0.084	0.090	0.090	0.60	6.67
11	0.156	0.133	0.143	0.144	1.15	8.01
12	0.146	0.122	0.132	0.133	1.21	9.04
13	0.696	0.703	0.700	0.700	0.35	0.50
14	0.423	0.448	0.427	0.433	1.34	3.10
15	0.141	0.141	0.139	0.140	0.12	0.82
16	0.111	0.099	0.100	0.103	0.67	6.44

**Table 2:** Detection results of inter-batch repeatability.

Number	Batches			Mean	SD (%)	CV (%)
	1	2	3			
1	0.612	0.649	0.607	0.623	2.29	3.68
2	0.521	0.490	0.535	0.515	2.30	4.47
3	0.562	0.560	0.520	0.547	2.37	4.33
4	0.729	0.701	0.781	0.737	4.06	5.51
5	0.520	0.545	0.551	0.539	1.64	3.05
6	0.735	0.703	0.776	0.738	3.66	4.96
7	0.386	0.369	0.342	0.366	2.22	6.07
8	0.631	0.589	0.579	0.600	2.76	4.60
9	0.388	0.346	0.352	0.362	2.27	6.28
10	0.309	0.300	0.347	0.319	2.49	7.83
11	0.683	0.658	0.730	0.690	3.66	5.30
12	0.067	0.064	0.064	0.065	0.17	2.66
13	1.257	1.159	1.241	1.219	5.26	4.31
14	0.132	0.140	0.159	0.144	1.39	9.65
15	0.080	0.083	0.085	0.083	0.25	3.04
16	0.161	0.169	0.151	0.160	0.90	5.62

**Table 3:** Detection results for the specificity of ELISA.

Serum	PDCoV	PEDV	TGEV	PRRSV	CSFV	PCV2
S/P	0.98	0.22	0.27	0.10	0.08	0.12
Result	+	−	−	−	−	−

**Table 4:** Sampling results of clinical sera in different regions of Zhejiang from 2021 to 2024.

	Year	2021	2022	2023	2024	Total
Region						
Hangzhou		16	32	18	-	66
Jinhua		16	40	18	33	107
Quzhou		16	-	32	37	85
Lishui		16	35	18	20	89
Shaoxing		17	-	27	-	44
Wenzhou		19	-	36	30	85
Huzhou		46	-	-	-	46
Taizhou		-	-	25	-	25
Ningbo		-	-	38	15	53
Total		146	107	212	135	600

**Table 5:** Positive rate of antibodies to PDCoV in pig herds in different regions of Zhejiang.

Cities	Serum Number	Positive Number	Positive Rate (%)
Hangzhou	66	20	30.3
Jinhua	107	11	10.3
Quzhou	85	18	21.2
Lishui	89	38	42.7
Shaoxing	44	5	11.4
Wenzhou	85	13	15.3
Taizhou	25	2	8.0
Huzhou	46	5	10.8
Ningbo	53	4	7.5
Total	600	116	19.3

**Table 6:** Positive rate of antibodies to PDCoV in pig herds from 2021 to 2024.

Year	2021	2022	2023	2024
Serum count	146	107	212	135
Positive number	26	33	21	36
Positive rate (%)	17.8	30.8	9.9	26.7

## Data Availability

The data presented in this study are available on request from the corresponding author due to regulations and agreements governing uncharacterized animal sample resources.

## Supplementary Materials

The following supporting information can be downloaded at https://www.mdpi.com/article/10.3390/vetsci13010012/s1, Figure S1: The supernatant and precipitate of the recombinant PDCoV N protein were detected by Western blot. Figure S2: Western blot analysis was performed using the N proteins of three porcine enteric coro-naviruses and control proteins as antigens.
