# Supplementary figures and images for: Establishment and Application of PDCoV Antibody Indirect ELISA Detection Method Based on N Protein
Source: Vet Sci. 2025 Dec 22;13(1):12. doi: 10.3390/vetsci13010012 (PMC12846618; doi:10.3390/vetsci13010012)

Figure S1

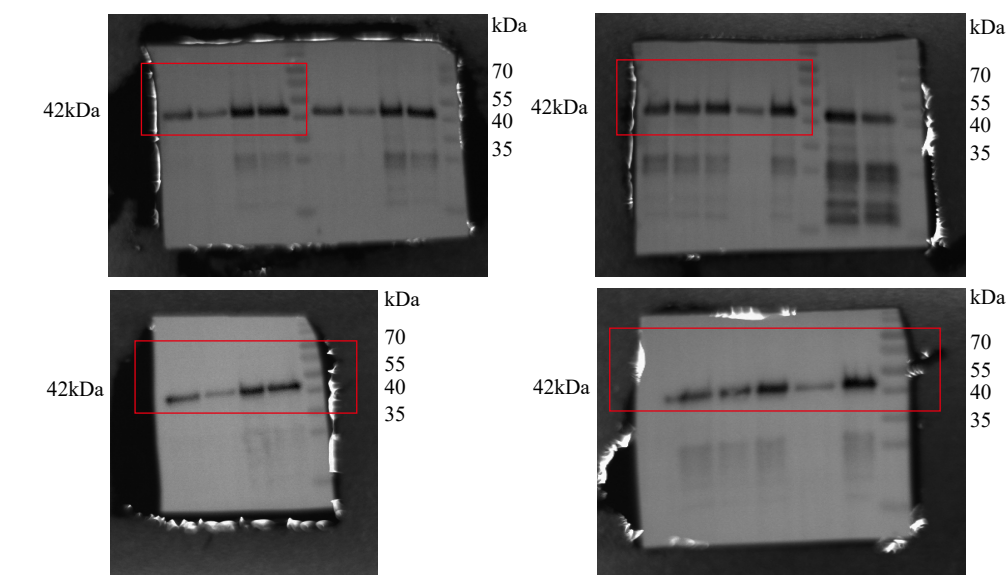

Figure S2

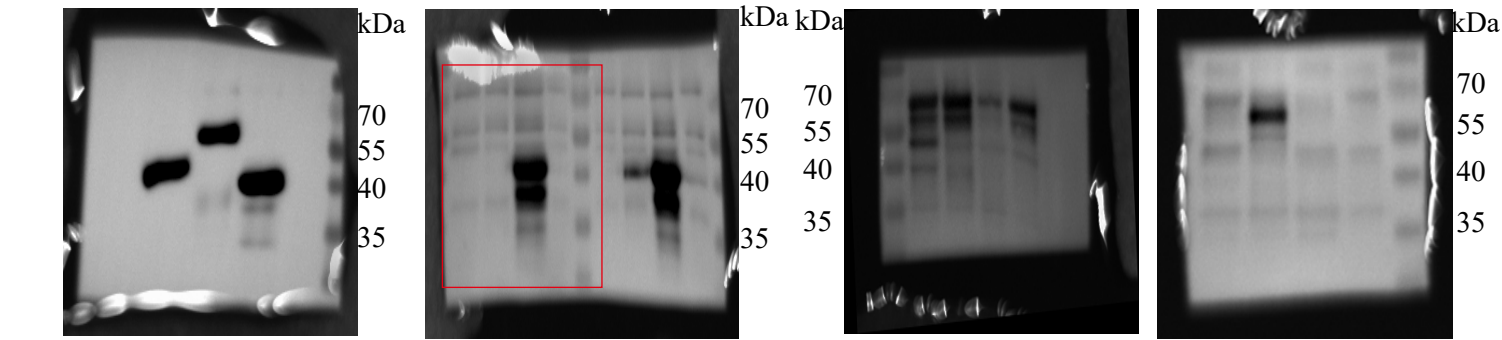

Supplement: Supplementary file 1 [file vetsci-13-00012-s001.zip › vetsci-4020742-supplementary.pdf]
